# The Influence of Glycemic Control Over Post-extraction Healing in Diabetic Patients

**DOI:** 10.7759/cureus.70998

**Published:** 2024-10-07

**Authors:** Jegan M, A Saneem Ahamed, Vijaya Lakshmi G

**Affiliations:** 1 Dentistry, Priyadarshini Dental College and Hospital, Chennai, IND; 2 Oral and Maxillofacial Surgery, Priyadarshini Dental College and Hospital, Chennai, IND

**Keywords:** dental extraction, diabetes mellitus, glycemic levels, post-extraction complications, wound healing

## Abstract

Background

Diabetes mellitus hinders wound healing after tooth extractions due to hyperglycemia, causing complications like infections and delayed recovery. The precise role of glycemic control in healing remains uncertain. This study aims to clarify it by comparing outcomes in patients with well-controlled and poorly controlled blood sugar levels.

Material and methods

This prospective observational study at Priyadarshini Dental College and Hospital (October-November 2023) involved 100 insulin-dependent diabetic patients (n = 100) requiring dental extractions, divided into group A with random blood sugar (RBS) ≤150 mg/dL (n = 50) and group B with RBS between 151 and 240 mg/dL (n = 50). Inclusion criteria included fully erupted teeth in patients aged 18 or older, while exclusion criteria comprised those on antibiotics, with systemic immunodeficiency, undergoing chemotherapy, or with active infections. Preoperative RBS and post-extraction wound dimensions were recorded, and clinical outcomes, such as pain, bleeding, infection, dry socket, and tissue color changes, were assessed on days 1, 7, and 14. Ethical approval was obtained from the Institutional Ethical Committee (IEC-PDCH5/4-2023) and the Indian Council of Medical Research (ICMR Reference ID: 2023-14931). Data were analyzed using chi-square and independent sample t-tests.

Results

The findings suggest that higher glycemic levels (151-240 mg/dL) are associated with increased pain and complications but do not significantly affect healing outcomes compared to levels ≤150 mg/dL. Group A shows better healing status, while group B has a higher incidence of moderate pain and bleeding.

Conclusion

This study indicates that glycemic levels between 151 and 240 mg/dL have no significant impact on post-extraction healing compared to levels ≤150 mg/dL in diabetic patients. Elevated blood sugar within this range may not increase the risk of delayed healing or complications. However, managing blood sugar levels is still critical, as uncontrolled diabetes can lead to adverse outcomes. Further research is required to determine the ideal glycemic range for optimal healing in diabetic patients.

## Introduction

Diabetes mellitus is acknowledged as a significant systemic disorder that is increasingly affecting a growing population. It primarily leads to various complications, including retinopathy, nephropathy, obesity, and impaired wound healing [1]. Persistent hyperglycemia in diabetes adversely impacts all body tissues, leading to increased morbidity and mortality [2]. Over 90% of diabetic patients exhibit oral manifestations [3]. Individuals with diabetes are particularly vulnerable to developing oral complications, especially periodontal diseases. Oral infections may also negatively affect glycemic control [4]. Moreover, the presence of oral diseases can exacerbate the systemic manifestations of diabetes mellitus [5]. It is widely recognized that wounds and soft tissue injuries in individuals with diabetes mellitus often exhibit poor healing [6].

Disruption of the healing process in extraction sockets, due to compromised systemic function and various risk factors, can result in delayed healing, wound dehiscence, infection, and potentially severe outcomes. The healing process, histologically, consists of four stages: the blood clot phase, the inflammatory phase with granulation tissue formation, the proliferative phase leading to woven bone formation, and the modeling and remodeling phase [3]. Effective healing of bone injuries is essential for restoring function and preventing complications. This process is influenced by several factors, which can either delay or enhance healing. These factors include tissue type, wound condition and location, microbial status, vascular supply, and local and systemic factors. Systemic factors, such as the proliferation and differentiation of growth factors, also play a critical role in the healing process [7]. Proper management of these factors is crucial for achieving optimal healing outcomes.

The multifactorial etiology of delayed wound healing in uncontrolled diabetes remains a subject of ongoing debate [8]. Delays in healing may be attributed to various factors. Insulin significantly affects healing by influencing molecules such as insulin-like growth factors, transforming growth factor β3, and bone morphogenetic proteins. However, elevated blood sugar levels in diabetic individuals can impair the immune system, reduce macrophage function, and increase the risk of infection. Additionally, elevated ketone levels and decreased nitric oxide (NO) levels may further hinder healing by constricting blood vessels and limiting oxygen and nutrient delivery [7].

Disruption of the healing process can lead to delayed healing, wound dehiscence, infection, and potentially life-threatening outcomes [9]. Recent research highlights that diabetes mellitus affects virtually every tissue in the body, either directly or indirectly, through its late complications. Dentists frequently encounter diabetic patients in their practice, making it necessary to assess the diabetic status of patients before performing dental extractions. This study is designed to evaluate whether a patient’s diabetic status affects wound healing in a post-extraction socket. This is a crucial aspect in preventing socket infection and optimizing post-operative care for diabetic patients. The aim of this study is to determine whether glycemic control influences healing after tooth extractions.

## Materials and methods

Ethical approval

This study received ethical clearance from the Institutional Ethical Committee (IEC-PDCH5/4-2023) and was approved by the Indian Council of Medical Research (ICMR Reference ID: 2023-14931).

Study design

This prospective observational study was conducted at the Oral and Maxillofacial Surgery Department of Priyadarshini Dental College and Hospital, Chennai, India, between October 2023 and November 2023. Ethical clearance was obtained from the Institutional Ethical Committee. The study included 100 patients (n = 100) with insulin-dependent diabetes mellitus who required dental extractions. The patients were categorized into two groups: group A (n = 50) consisted of patients with a preoperative random blood sugar (RBS) level ≤150 mg/dL, while group B (n = 50) included patients with RBS levels between 151 and 240 mg/dL.

Inclusion criteria consisted of patients with a fully erupted tooth suitable for extraction, aged 18 years or older. Exclusion criteria included patients on antibiotic or steroid use, those with systemic immunodeficiency, patients undergoing chemotherapy or radiation therapy, and those with third molars.

Data collection

**Figure 1:**
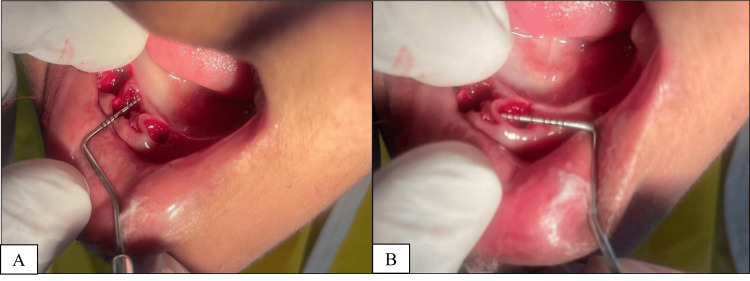
(A) Mesiodistal width measurement demonstrated with a periodontal probe and (B) buccolingual width measurement demonstrated with a periodontal probe

Preoperative RBS levels were measured for all participants. Patients undergoing extraction provided informed consent prior to the procedure. Following extraction, mesiodistal and buccolingual wound widths were measured using periodontal probes and dividers, as illustrated in (Figures 1A, 1B).

**Figure 2:**
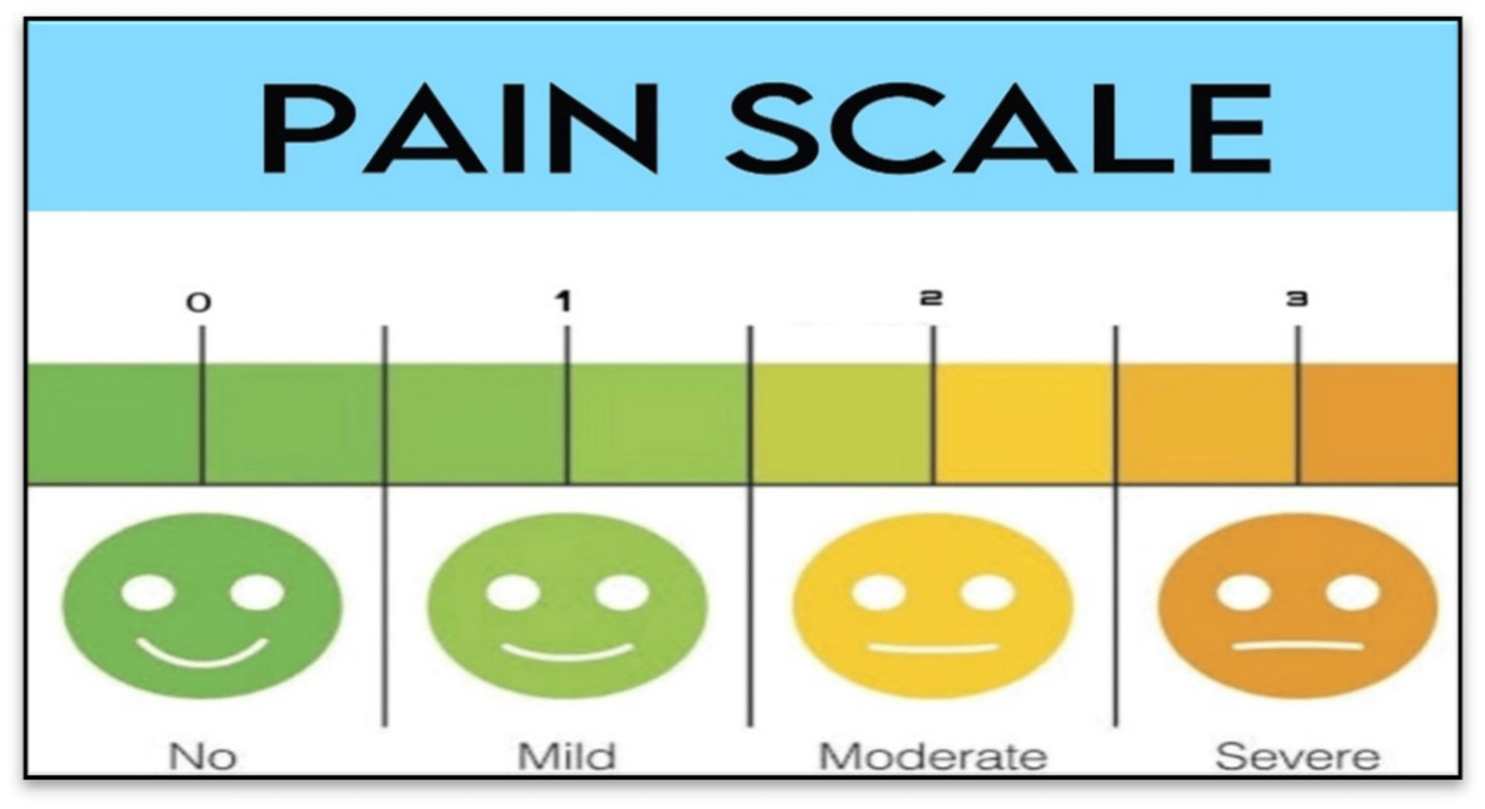
Verbal pain rating scale

Clinical criteria such as pain, postoperative bleeding, infection, dry socket, and tissue color change were assessed on the first, seventh, and 14th days. A verbal pain rating scale (0-3) was used to evaluate postoperative pain, with the corresponding pain scale depicted in Figure 2.

Statistical analysis

Postoperative infection was identified by the presence of pus and purulent discharge. Alveolar osteitis was defined by meeting two criteria: exposed bone in the socket and a foul odor or taste in the mouth. The collected data were analyzed statistically using the chi-square test and an independent sample t-test to evaluate all values.

## Results

Demographic and RBS analysis

Data were collected from two groups in the study. Group B had an older average age (55.48 years) compared to group A (50.38 years). Both groups showed a similar distribution in terms of sex, with a slightly higher percentage of females in group B (60%) compared to group A (52%). In terms of RBS levels, group B demonstrated significantly higher levels, with an average of 196.20 mg/dL, while group A had an average of 124.66 mg/dL. Group A’s RBS levels remained below or equal to 150 mg/dL, whereas group B’s RBS levels ranged between 151 and 240 mg/dL (Table 1).

**Table 1:** Demographic and random blood sugar (RBS) analysis

S.No.	Parameter	Group A	Group B
1	Age (mean ± SD)	50.38 ± 12.771	55.48 ± 11.070
2	Sex (M/F)	24:26 (48%:52%)	20:30 (40%:60%)
3	RBS (mean ± SD)	124.66 ± 15.923 (≤150 mg/dL)	196.20 ± 31.362 (151-240 mg/dL)

Pain rating analysis

**Figure 3:**
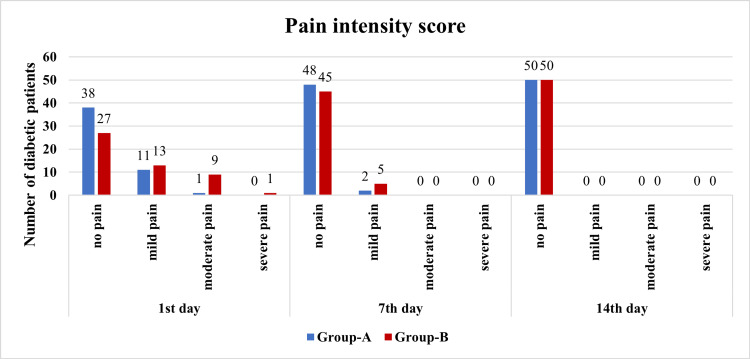
Pain intensity score in diabetic patients post-tooth extraction

Data were collected on pain levels experienced by participants in both groups on the first, seventh, and 14th day post-tooth extraction. Participants were categorized based on their pain intensity: no pain, mild pain, moderate pain, and severe pain. Group A had more participants reporting no pain compared to group B, with a statistically significant difference (P = 0.002). There was no significant difference between the groups in the occurrence of mild pain. However, group B had more participants experiencing moderate pain compared to group A, with a highly significant difference (P < 0.001). There was no significant difference in the occurrence of severe pain between the two groups. The pain intensity scores are further illustrated in graphical format (Figure 3).

For a detailed analysis of pain ratings and participant numbers, refer to Table 2.

**Table 2:** Comparison of the pain rating scores using a four-point verbal rating scale (VRS)

Time Interval	Pain rating scale	Group A (≤150 mg/dL)	Group B (151-240 mg/dL)	P-value
First day	No pain	38	27	0.024
	Mild pain	11	13	
	Moderate pain	1	9	
	Severe pain	0	1	
Seventh day	No pain	48	45	0.240
	Mild pain	2	5	
	Moderate pain	0	0	
	Severe pain	0	0	
14th day	No pain	50	50	<0.001
	Mild pain	0	0	
	Moderate pain	0	0	
	Severe pain	0	0	

This study collected data on complications experienced by participants in both groups following dental procedures. The analysis encompassed the number of participants reporting swelling, bleeding, infection, dry socket, and changes in tissue coloration on the first, seventh, and 14th days (Table 3).

**Table 3:** Comparison of dental complications between two groups on the first, seventh, & 14th day after tooth extraction

Time interval	Complications	Group A (<150 mg/dL)	Group B (150-240 mg/dL)	P-value
First day	Swelling	0	1	0.315
	Bleeding	6	15	0.027
	Infection	0	1	0.315
	Dry socket	0	0	<0.001
	Tissue color changes	0	0	<0.001
Seventh day	Swelling	0	0	<0.001
	Bleeding	0	0	<0.001
	Infection	1	2	0.558
	Dry socket	1	3	0.307
	Tissue color changes	0	1	0.315
14th day	Swelling	0	0	<0.001
	Bleeding	0	0	<0.001
	Infection	0	0	<0.001
	Dry socket	1	0	0.315
	Tissue color changes	1	2	0.558

Group B demonstrated a higher incidence of bleeding on the first day, with a statistically significant difference (P = 0.027). Furthermore, group B exhibited a trend toward increased swelling (P = 0.315) and infection (P = 0.558), although these differences did not reach statistical significance. In contrast, group A reported a significantly higher number of dry socket occurrences on the first day (P < 0.001). No significant differences in tissue color alterations were observed on the seventh day (P = 0.315). 

Comparative analysis of wound sizes

Data were collected on the wound sizes of participants in both group A and group B at three intervals: the first, seventh, and 14th days following the procedure. Wound sizes were measured in terms of mesiodistal and buccolingual widths, and the results are presented along with standard deviations (SD) in Table 4.

**Table 4:** Comparison of wound size between groups on the first, seventh, & 14th day after tooth extraction

Wound size	Time interval	Group A, mean (SD)	Group B, mean (SD)	P-value	95% Confidence interval of the difference	
					Lower	Upper
Mesiodistal width	Firstday	7.94 (1.889)	7.64 (1.967)	0.438	-0.465	1.065
	Seventh day	5.88 (1.814)	5.80 (2.129)	0.840	-0.705	0.865
	14th day	3.74 (1.536)	3.86 (1.750)	0.716	-0.773	0.533
Buccolingual width	Firstday	8.60 (2.914)	7.96 (2.740)	0.261	-0.483	1.763
	Seventh day	6.46 (2.533)	5.92 (2.481)	0.284	-0.455	1.535
	14th day	3.72 (1.874)	3.30 (2.003)	0.282	-0.350	1.190

Statistical analysis was performed to assess the significance of differences between the two groups. For mesiodistal width on the first day, no statistically significant difference was observed (P = 0.438, 95% CI [−0.465, 1.065]), and similarly for buccolingual width (P = 0.261, 95% CI [−0.483, 1.763]). On the seventh day, there were also no significant differences between the groups for mesiodistal width (P = 0.840, 95% CI [−0.705, 0.865]) or buccolingual width (P = 0.284, 95% CI [−0.455, 1.535]). On the 14th day, the trend remained consistent, with no significant differences in mesiodistal width (P = 0.716, 95% CI [−0.773, 0.533]) or buccolingual width (P = 0.282, 95% CI [−0.350, 1.190]). The graphical comparison of wound sizes across these time points is provided in Figure 5.

**Figure 4:**
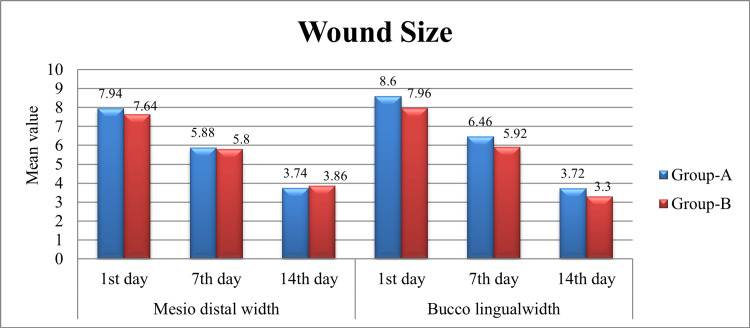
Comparison of wound size between groups on the first, seventh, and 14th day after tooth extraction, highlighting healing differences

Healing status of extraction sockets

Data were collected on the healing status of extraction sockets in two groups: group A and group B. Group A exhibited 44 cases of normal healing, accounting for 88% of participants, while six participants (12%) experienced abnormal healing. In contrast, group B had 35 cases (70%) of normal healing and 15 cases (30%) of abnormal healing. The statistical analysis using the chi-square test revealed a chi-square value of 4.82, with a P-value of less than 0.05, indicating a statistically significant difference in healing outcomes between the two groups (Table 5).

**Table 5:** Comparison of healing status in extraction sockets between group A and group B

Groups	Status of the extraction socket				Chi-square value	P-value
	Normal healing		Abnormal healing		4.82	<0.05
	Count	%	Count	%		
Group A	44	88%	6	12%		
Group B	35	70%	15	30%		

## Discussion

Diabetes is recognized as a significant risk factor for a variety of health conditions [1]. This study aimed to evaluate the impact of glycemic control on post-extraction healing in diabetic patients. Our findings indicate no statistically significant difference in healing outcomes between patients with well-controlled blood sugar levels (group A) and those with poorer glycemic control (group B). These results suggest that factors such as glycemic control may not significantly influence oral wound healing as formerly hypothesized [7].

This observation aligns with prior research, such as Aronovich et al., which demonstrated no statistically significant differences in healing outcomes between well-controlled and poorly controlled diabetic patients following tooth extractions, indicating comparable epithelialization rates [10]. Similarly, Muzaffar et al. conducted a study involving 100 diabetic patients, revealing that 75% achieved wound healing within 14 days post-extraction, with no significant delays observed in those with poor glycemic control [7]. Collectively, these studies indicate that glycemic control may not play a crucial role in post-tooth extraction healing among diabetic patients [7]. However, our study further contributes to this body of knowledge by specifically focusing on key post-extraction parameters, including pain, swelling, infection, dry socket, and tissue color changes.

Although our findings did not reveal statistically significant variances between the cohorts concerning glycemic regulation, it is imperative to acknowledge the clinical ramifications of these outcomes, with an emphasis on demographic variables, such as the older average age in group B and a marginally higher proportion of females, may contribute to the observed discrepancies in healing outcomes. This consideration is vital for elucidating the clinical implications of these findings.

The healing process of a tooth extraction socket is intricate, involving the repair and regeneration of tissue, [11] and the lack of a definitive correlation between glycemic indices and wound healing suggests that extrinsic and intrinsic factors may exert a more substantial influence on postoperative recuperation. Recent advancements in wound healing research have deepened the understanding of the complex interplay between diabetes and its physiological, inflammatory, immunological, endocrine, and neurological mechanisms, alongside the involvement of microRNAs (miRNAs) in the healing of extracted tooth sockets. The persistence of delayed wound healing in diabetic patients is predominantly associated with aberrant cellular functions and the dysregulation of crucial growth factors and cytokines necessary for the proper orchestration of the healing process, as evidenced by contemporary studies [3].

Interestingly, despite no significant statistical difference, group B (with higher RBS levels) exhibited a slightly higher occurrence of dry socket and infection. In this study, the dry socket was managed through saline irrigation and the application of a zinc oxide eugenol pack. Although these findings did not achieve statistical significance, they highlight the need for further research to explore whether other patient-specific factors or surgical protocols could contribute to these complications. This underscores the potential value of investigating individualized patient management strategies to enhance healing outcomes in diabetic patients following dental extractions.

Analogous findings were reported in the study conducted by Fernandes et al., which elucidated that although healing frequencies exhibited variability across different age demographics, glycemic control did not emerge as a statistically significant determinant of overall healing outcomes [4]. This observation underscores the intricate interplay of multiple factors that may concurrently influence post-extraction recovery, indicating that age-related physiological changes and other systemic variables could play a pivotal role in the healing process.

This study enhances the understanding of the impact of glycemic control on post-extraction healing in diabetic patients. By focusing on key parameters such as pain, swelling, and infection, the research underscores the complex interplay of various factors influencing healing outcomes. The extractions were performed by a single clinician to minimize variability and maintain consistency in technique, with assessments of healing progression conducted over a two-week period to effectively evaluate tissue recovery. Despite these merits, this study has limitations, including the short-term nature of the study, a relatively small sample size, and its conduction in a specific time and place, which may affect the generalizability of the findings.

## Conclusions

Insights from this study elucidate the ramifications of glycemic control on post-extraction healing in diabetic patients. Although discernible differences in healing outcomes exist between the groups, the absence of statistically significant P-values across several parameters suggests that glycemic control may not be the exclusive determinant influencing these outcomes. These findings, congruent with the short-term nature of the investigation, underscore the necessity for further research with larger sample sizes and extended follow-up durations to comprehensively elucidate the multifactorial dynamics of post-extraction healing in diabetic populations.
